# Autochthonous tick-borne lymphadenopathy (TIBOLA) in a 3-year-old child caused by *Rickettsia slovaca* in southwestern Germany

**DOI:** 10.1007/s15010-026-02899-4

**Published:** 2026-07-31

**Authors:** Anne Isaak, Dennis Tappe, Alexandra Veit, Thomas Theo Brehm

**Affiliations:** 1 Praxis Christiane Krysl, Bretzfeld, Germany; 2 Bernhard Nocht Institute for Tropical Medicine, National Reference Centre for Tropical Pathogens, Hamburg, Germany; 3 Division of Infectious Diseases, I. Department of Medicine, University Medical Center Hamburg-Eppendorf, Martinistraße 52, 20246 Hamburg, Germany; 4 Clinical Infectious Diseases, Research Center Borstel, Leibniz Lung Center, Borstel, Germany; 5 Clinical Tuberculosis Unit, German Center for Infection Research (DZIF), Hamburg- Lübeck-Borstel-Riems, Germany

**Keywords:** Tick-borne lymphadenopathy, TIBOLA, Rickettsiosis, Dermacentor tick, Rickettsia slovaca, Germany

In March 2026, a 3-year-old child from Baden-Württemberg, southwestern Germany, presented with fever of up 39.0 °C, painful cervical and occipital lymphadenopathy, a few small erythematous skin lesions on the plantar aspects of the feet, and a necrotic eschar on the scalp following a recalled tick bite, consistent with tick-borne lymphadenopathy (TIBOLA) (Fig. 1). PCR testing and sequencing of rickettsial *ompB* genes from the eschar confirmed *Rickettsia slovaca* as causative pathogen. Serological testing performed four weeks after the initial presentation demonstrated IgM and IgG antibodies against spotted fever group rickettsiae using heterologous *R. africae* antigens in a validated *in-house* indirect immunofluorescence assay (IgM 1:40, IgG 1:80; cut-off as determined with 20 healthy blood donors, 1:40; sensitivity 78%, specificity for spotted fevers in a group containing also typhus group rickettsioses 85%), consistent with recent rickettsial infection. As no serum sample was obtained at symptom onset, seroconversion could not be demonstrated. Given the mild clinical course and the patient’s young age, treatment with oral azithromycin was initiated at a dose of 10 mg/kg body weight once daily on day 1, followed by 5 mg/kg once daily on days 2–5, with gradual clinical improvement becoming apparent only on the final day of treatment.

TIBOLA, also called scalp eschar and neck lymph adenopathy after a tick bite (SENLAT), is a spotted fever group rickettsiosis transmitted by *Dermacentor* ticks, most commonly caused by *Rickettsia slovaca*, and predominantly affects women and children. This case represents only the second published autochthonous TIBOLA case from Germany, following a report from the same region in 2011 [1] while imported cases had also been described [2]. Although *Rickettsia slovaca* is known to occur in southwestern Germany due to the established distribution of its *Dermacentor* tick vectors, TIBOLA remains an underrecognized clinical syndrome and may therefore be overlooked in routine clinical practice. Increasing case numbers reported from neighboring regions suggest that TIBOLA may be an emerging disease, potentially reflecting both expanding *Dermacentor* tick populations and improved clinical awareness and diagnostic testing [3, 4]. The classic triad of scalp eschar, cervical lymphadenopathy, and fever after tick exposure during cooler months should prompt consideration of TIBOLA [5]. In spotted fever and typhus group rickettsioses, antibodies become detectable after approximately 10–14 days and may not always reach high titers, leading to relatively low sensitivity. In contrast, PCR testing from eschars in spotted fever group rickettsiosis is nearly uniformly positive. Doxycycline remains the treatment of choice for tick-borne rickettsial diseases in patients of all ages, including children under 8 years, as short courses have not been shown to cause dental staining [6]. It should therefore be used in all severe cases, including in pediatric patients.

This case underscores TIBOLA as a potential emerging infectious disease in Central Europe, warranting increased clinical awareness and surveillance.

**Fig. 1:**
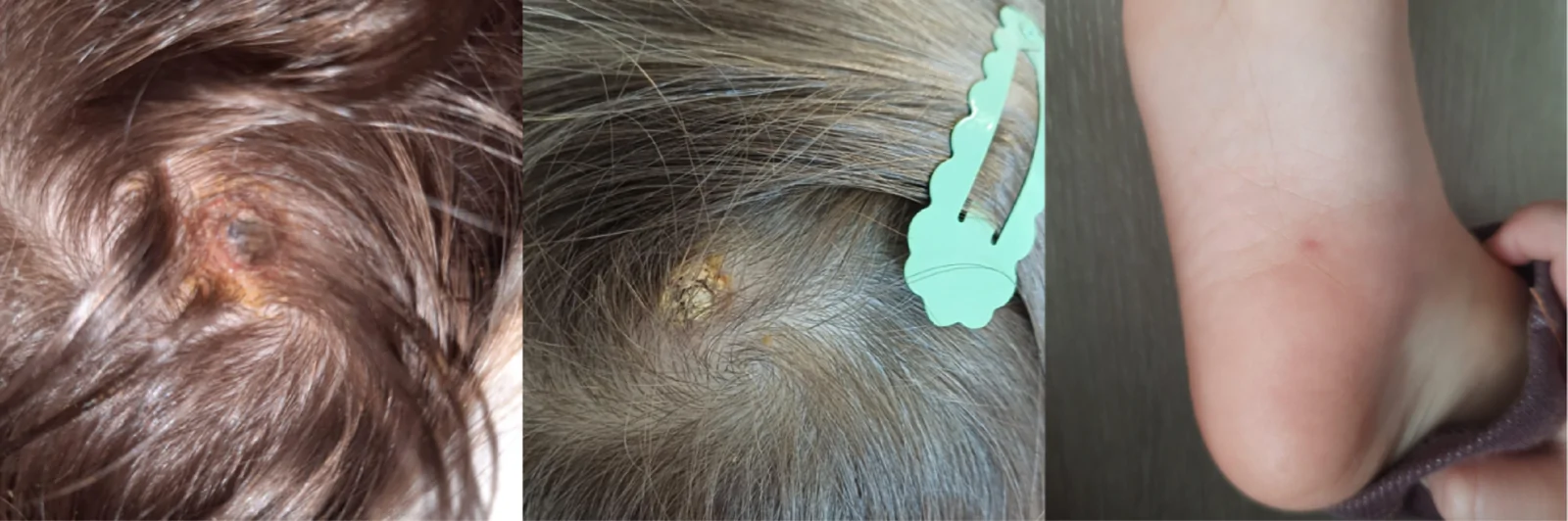
Characteristic TIBOLA eschar located on the scalp at presentation (left) and four weeks later (middle), and a representative erythematous skin lesion on the plantar aspect of the foot (right)

## Data Availability

No datasets were generated or analysed during the current study.
